# Chronic kidney disease outcomes in patients treated with immune checkpoint inhibitors

**DOI:** 10.1093/ckj/sfag224

**Published:** 2026-07-11

**Authors:** Nabil Abu-Amer, Amir Givon, Nirit Agay, Havi Murad, Noi Horesh, Margarita Kunin, Orit Erman, Adva Vaisman, Haim Mayan, Pazit Beckerman, Ronen Loebstein

**Affiliations:** Institute of Nephrology and Hypertension, Sheba Medical Center, Tel Hashomer, Israel; Gray Faculty of Medical and Health Sciences, Tel Aviv University, Tel Aviv, Israel; Gray Faculty of Medical and Health Sciences, Tel Aviv University, Tel Aviv, Israel; Institute of Clinical Pharmacology and Toxicology, Sheba Medical Center, Tel Hashomer, Israel; Biostatistics & Biomathematics Unit, Data & Analytics Division, Sheba Medical Center, Tel Hashomer, Israel; Biostatistics & Biomathematics Unit, Data & Analytics Division, Sheba Medical Center, Tel Hashomer, Israel; Department of Internal medicine C, Sheba Medical Center, Tel Hashomer, Israel; Institute of Nephrology and Hypertension, Sheba Medical Center, Tel Hashomer, Israel; Gray Faculty of Medical and Health Sciences, Tel Aviv University, Tel Aviv, Israel; Institute of Nephrology and Hypertension, Sheba Medical Center, Tel Hashomer, Israel; Gray Faculty of Medical and Health Sciences, Tel Aviv University, Tel Aviv, Israel; Institute of Nephrology and Hypertension, Sheba Medical Center, Tel Hashomer, Israel; Gray Faculty of Medical and Health Sciences, Tel Aviv University, Tel Aviv, Israel; Gray Faculty of Medical and Health Sciences, Tel Aviv University, Tel Aviv, Israel; Institute of Clinical Pharmacology and Toxicology, Sheba Medical Center, Tel Hashomer, Israel; Institute of Nephrology and Hypertension, Sheba Medical Center, Tel Hashomer, Israel; Gray Faculty of Medical and Health Sciences, Tel Aviv University, Tel Aviv, Israel; Gray Faculty of Medical and Health Sciences, Tel Aviv University, Tel Aviv, Israel; Institute of Clinical Pharmacology and Toxicology, Sheba Medical Center, Tel Hashomer, Israel

**Keywords:** cancer, chemotherapy, chronic kidney disease, ESKD, immune checkpoint inhibitors

## Abstract

**Background:**

Immune checkpoint inhibitors (ICIs) are increasingly used in oncology, but long-term kidney outcomes in patients with pre-existing CKD (chronic kidney disease) are not well understood. We assessed CKD progression in patients with a baseline eGFR of 15–60 ml/min/1.73 m² who were treated with ICIs, comparing outcomes with those receiving chemotherapy.

**Methods:**

We conducted a retrospective cohort study at Sheba Medical Center between 2015 and 2024. The primary outcome was a composite of new-onset end-stage kidney disease, initiation of kidney replacement therapy, or a >30% eGFR decline for at least 90 days. The secondary outcome was time to the first composite event. We used a Cox model with time-varying exposure and a propensity score-matched, new-user active comparator analysis to minimize selection bias.

**Results:**

Of 3468 patients treated with ICIs, 885 had a baseline of eGFR 15–60 ml/min/1.73 m²; 208 met the inclusion criteria for CKD progression analysis. The composite outcome occurred in 22% of patients, with a shorter time-to-event in CKD stage 4 than in CKD stage 3 (27 vs. 83 months; *P* = 0.003). Factors associated with CKD progression included hydronephrosis (HR 5.6), smoking (HR 2.09), higher eosinophil counts (HR 4.8), and higher baseline creatinine (HR 3.0). Higher hemoglobin reduced the risk by 12%. ICI exposure was not associated with an increased risk compared with chemotherapy in the time-varying analysis (HR 0.83) or the matched active comparator (HR 0.81). These findings remained consistent in sensitivity analyses excluding patients with genitourinary malignancies.

**Conclusions:**

Among patients with eGFR 15–60 ml/min/1.73 m², systemic anticancer therapy carries a substantial risk of renal decline; however, ICIs do not increase the risk of CKD progression compared to chemotherapy. Outcomes are predominantly driven by baseline renal impairment and individual risk factors, highlighting the necessity of close renal monitoring rather than avoiding ICIs in this vulnerable population.

KEY LEARNING POINTS **What was known:**

- Patients with pre-existing chronic kidney disease (CKD) are often underrepresented in immune checkpoint inhibitor (ICI) trials, and long-term kidney outcomes in this group remain uncertain.

**This study adds:**

- In this real-world cohort, ICIs were associated with a high risk of CKD progression (22%) but not greater than chemotherapy; progression was mainly associated with baseline CKD severity and other clinical risk factors.

**Potential impact:**

- These findings support the use of ICIs in patients with CKD when accompanied by regular kidney monitoring and active management of modifiable renal risk factors.

## INTRODUCTION

Immune checkpoint inhibitors (ICIs) have revolutionized the oncology landscape, significantly improving outcomes across various malignancies by targeting cytotoxic T-lymphocyte-associated protein 4 (CTLA-4), programmed death 1 (PD-1), and programmed death-ligand 1 (PD-L1) pathways [1–4]. Their increasing use has also raised awareness of immune-related adverse events (irAEs), including kidney toxicity [5–12]. Although immune-mediated interstitial nephritis and acute kidney injury (AKI) are well documented, the long-term effects of ICIs on kidney function in patients with pre-existing chronic kidney disease (CKD) remain not fully understood [13, 14].

Patients with eGFR <60 ml/min/1.73 m² are at higher risk of CKD progression and treatment-related complications [15–17]. Despite representing a substantial and highly vulnerable clinical demographic, these patients are systematically excluded from pivotal ICI clinical trials. This exclusion has created a profound gap in evidence-based oncology, as the complex interplay between systemic anticancer therapy, immune-related adverse events, and baseline renal impairment makes management more complex and challenging.

To address this critical paucity of data, this study aims to evaluate key knowledge gaps regarding the long-term kidney risks associated with ICIs exposure in a real-world cohort of patients with pre-existing CKD. The results could help healthcare providers better predict and manage CKD progression in this vulnerable group, and facilitate a safer balance between maximizing the oncological benefits of immunotherapy and mitigating the risk of irreversible kidney decline.

## MATERIALS AND METHODS

### Study design

We conducted a real-world retrospective cohort study of adult patients (≥18 years) with solid malignancies and baseline eGFR of 15-60 ml/min/1.73 m² who were treated with ICIs and/or chemotherapy at Sheba Medical Center from 2015 to 2024, all of whom had kidney function follow-up. First, we analyzed the ICI-treated cohort separately, then compared kidney outcomes between ICI and chemotherapy exposure periods. Furthermore, we compared the risk of kidney function decline during both the ICI and chemotherapy periods to assess whether deterioration was related to treatment type or to baseline patient risk.

Data were collected from institutional electronic databases containing demographic information, cancer type, treatment exposures, comorbidities, laboratory results, chronic medications, nephrotoxic agents, imaging findings, and urinalysis data. Mortality data were obtained from the National Ministry of the Interior Population Registry through December 2024. The study was approved by the Sheba Medical Center Institutional Review Board (SMC-6545-19), and the requirement for informed consent was waived due to the study’s retrospective nature.

### Population

We included adults with solid tumors who were treated with an ICI-based regimen. ICIs were administered as monotherapy (e.g. CTLA-4, PD-1, or PD-L1 inhibitors), in combination with other ICIs (e.g. CTLA-4 inhibitors across different ICI classes), or in combination with chemotherapy. Patients had an eGFR of 15–60 ml/min/1.73 m² at the start of treatment. The index date was the date the first systemic treatment was administered.

Baseline serum creatinine was defined as the average of two outpatient serum creatinine measurements taken within three months of each other and within the year before the index date. During follow-up, creatinine values were derived from routine pre-treatment blood testing, allowing continuous eGFR tracking.

We excluded patients younger than 18 years, those without two qualifying pre-index creatinine measurements, patients with baseline ESKD or prior kidney transplants, and those who survived <1 year after the index date. Follow-up was censored at the first composite outcome, the last available eGFR measurement, or death if it occurred within 1 month of the last eGFR measurement.

For the ICI-versus-chemotherapy comparison, the primary analysis included patients who were exposed to ICIs, chemotherapy, or both and survived for at least 1 year. A sensitivity analysis was limited to patients treated only with ICIs or chemotherapy who survived at least 1 year. The chemotherapy group was selected from a cohort of ∼14 000 patients treated with chemotherapy.

### Exposure definition

The study’s exposure group included patients with mild-to-severe CKD. eGFR was calculated using the 2021 CKD-EPI equation [18]. Baseline CKD was categorized as eGFR 45–59, 30–44, and 15–29 ml/min/1.73 m².

### Outcomes

The primary outcome was a composite of new-onset end-stage kidney disease (ESKD), initiation of kidney replacement therapy (KRT), or a sustained ≥30% decrease in eGFR from baseline for at least 90 days. This composite endpoint reflects the multidimensional nature of CKD progression and its serious clinical effects. This approach captures significant changes in kidney function over time and provides a comprehensive view of CKD outcomes in patients with baseline kidney impairment on ICIs. The secondary outcome was the time to the first composite renal event.

AKI was defined according to the KDIGO criteria as an increase in serum creatinine by ≥0.3 mg/dL within 48 h or to ≥1.5 times the baseline value. Given the retrospective nature of our institutional database, urine output criteria were not systematically available across the cohort and were therefore not utilized for the diagnosis of AKI.

### Statistical analysis

In the ICI-only cohort, we compared baseline characteristics based on the occurrence of the composite outcome. Time to the composite outcome was analyzed using Kaplan–Meier curves and the log-rank test for CKD stage 3 vs. stage 4. To account for death as a competing risk, we fitted univariable Fine and Gray models adjusted for age, sex, and baseline creatinine for each candidate variable. Then, we built a multivariable model, including variables with *P* < 0.20, using backward elimination.

For the treatment comparison, we used a time-varying exposure approach in which ICI exposure was coded as 0 before ICI initiation and 1 afterward, allowing patients to contribute person-time based on their actual treatment status and reducing immortal-time bias [19, 20]. Models were adjusted for age, sex, cancer type, and baseline eGFR. Death was treated as a competing risk using Fine and Gray methods.

Since genitourinary malignancies frequently cause mechanical urinary tract obstructions that could independently influence kidney outcomes, we repeated the primary analysis (time-varying exposure approach) as a sensitivity analysis by excluding patients with GU cancers from the model to ensure our findings were not disproportionately driven by post-renal obstructive nephropathy.

To rigorously compare the effects of ICIs vs. chemotherapy on kidney outcomes while minimizing selection bias, we conducted a secondary sensitivity analysis using a well-structured propensity score matching approach within a new-user active-comparator design. We include only treatment-naive patients: those initiating ICIs without prior chemotherapy (cases) and those starting chemotherapy without prior ICIs (controls). Patients who received ICIs and chemotherapy concurrently were excluded. This incident-new-user cohort design aimed to enhance internal validity by reducing bias and improving comparability between the treatment groups [21, 22].

For each case, we set the index date (cohort entry) to the date immunotherapy was initiated. We then created exposure sets by identifying eligible chemotherapy-treated controls who began treatment within ±60 days of the case’s index date. To be eligible for inclusion in an exposure set, the patient must not have experienced a kidney deterioration event or death prior to the index date. Within each exposure set, we estimated time-conditional propensity scores for initiating ICIs using conditional logistic regression. Covariates included age at the index date, cancer category, and CKD stage at the index date. For each ICI-treated case, one comparator control was selected via 1:1 matching with replacement based on propensity scores. Matching was performed separately within each exposure set using the PSMATCH procedure in SAS 9.4, employing greedy nearest-neighbor matching with a caliper of 0.25 standard deviations of the propensity score. To evaluate the positivity assumption, we used the “Region = treated” option in the PSMATCH procedure, limiting matching to controls with propensity scores within the range observed among treated cases. Finally, to assess differences in the risk of kidney deterioration between ICIs and chemotherapy users, a marginal Cox model was used, treating death as a competing risk. A robust sandwich covariance matrix estimate was used to account for the reuse of controls resulting from matching with replacement.

This approach yielded a matched cohort of ICI and chemotherapy initiators, enabling a direct and balanced comparison of renal outcomes between treatment groups.

## RESULTS

**Figure 1:**
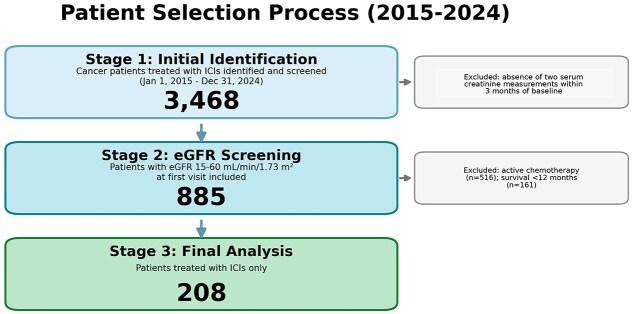
Study flow chart. A total of 3468 patients diagnosed with cancer and treated with ICIs between 1 January 2015 and 31 December 2024 were screened. Of these, 885 patients had eGFR 15–60 ml/min/1.73 m². After application of the study eligibility criteria, 208 patients treated with ICIs only were included in the ICI cohort analysis. **ICIs**- Immune checkpoint inhibitors; **eGFR**- estimated glomerular filtration rate.

Between 1 January 2015, and 31 December 2024, 3468 patients treated with ICIs were screened. Of these, 885 had a baseline eGFR of 15–60 ml/min/1.73 m², and 208 patients treated only with ICIs met the inclusion criteria for the ICI cohort analysis (Fig. 1).

In the ICI cohort, 68% were male, with a mean age of 74 ± 9 years, and the mean eGFR was 46 ± 10 ml/min/1.73 m². Most patients had CKD stage 3 (90%), and the most common cancer types were genitourinary malignancies (40%), melanoma (20%), and lung cancer (18%). Overall, 45 patients (22%) reached the composite kidney outcome.

The median age of patients who reached the composite outcome was 73.8 years (IQR, 69.09–76.7), and 68.9% (31 patients) were male. These patients had higher baseline serum creatinine, lower baseline eGFR, lower hemoglobin, and higher eosinophil counts than those who did not. They were also more likely to be smokers, have undergone major surgery, and have pre-index hydronephrosis on imaging (Table 1). AKI occurred in 42% (19/45) of patients who reached the composite outcome and in 29% (47/163) of those who did not (OR 1.8, 95% CI 0.91–3.57), but this association was not statistically significant (*P* = 0.09). Among the ICI cohort, 23.6% (49 patients) were receiving angiotensin-converting enzyme inhibitors (ACE-i), 33.7% (70 patients) were receiving angiotensin receptor blockers (ARBs), and 5.8% (12 patients) were receiving sodium-glucose cotransporter 2 inhibitors (SGLT2-i). Baseline use of these medications did not significantly differ between patients who reached the composite outcome and those who did not (*P* = 0.08 for ACE-i; *P* = 0.7 for ARBs; *P* = 0.7 for SGLT2-i).

**Table 1:** Baseline characteristics.

	Composite outcome		
Characteristic	No (*n* = 163)	Yes (*n* = 45)	*P*
Age (years), median (IQR)	74.5 (67.5–80.2)	73.8 (69.09–76.7)	0.3
Male, *n* (%)	110 (67.5)	31 (68.9)	0.9
**Cancer, *n* (%)**			
Breast	5 (3.1)	0 (0.0)	0.4
Digestive system	16 (9.8)	4 (8.9)	
GU system	61 (37.4)	25 (55.6)	
Lung	29 (17.8)	8 (17.8)	
Gynecologic	7 (4.3)	2 (4.4)	
Skin	35 (21.5)	6 (13.3)	
Head and neck	6 (3.7)	0 (0.0)	
Other	4 (2.4)	0 (0.0)	
**CKD stage, *n* (%)**			
3a	107 (65.6)	19 (42.2)	0.004
3b	45 (27.6)	17 (37.8)	
4	11 (6.7)	9 (20.0)	
eGFR at baseline (ml/min), median (IQR)	49.50 (41.3–54.9)	40.50 (32.2–54.6)	0.02
Creatinine at baseline (mg/dl), median (IQR)	1.41 (1.2–1.6)	1.51 (1.36–1.93)	0.008
EOS (k/µl), median (IQR)	0.13 (0.06–0.2)	0.19 (0.09–0.3)	0.02
LDH (U/l), median (IQR)	225.0 (184–271)	214.0 (178–272)	0.7
Albumin (g/dl), median (IQR)	3.80 (3.30–4.1)	3.60 (3.2–4)	0.9
HGB (g/dl), median (IQR)	11.64 (10.5–13.01)	10.55 (9.5–12.07)	0.007
WBC (k/µl), median (IQR)	7.50 (5.7–9.6)	7.70 (5.8–9.9)	0.5
CRP (mg/l), median (IQR)	16.08 (5.7–61)	18.29 (6.5–62.4)	0.6
Chronic diuretics, *n* (%)	48 (29.4)	15 (33.3)	0.6
Chronic NSAIDS, *n* (%)	6 (3.7)	1 (2.2)	0.6
Chronic ACE-i, *n* (%)	34 (20.9)	15 (33.3)	0.08
Chronic ARB, *n* (%)	56 (34.4)	14 (31.1)	0.7
Chronic SGLT2-i, *n* (%)	10 (6.1)	2 (4.4)	0.7
Steroid’s exposure, *n* (%)	22 (13.5)	7 (15.6)	0.7
Chronic PPI, *n* (%)	72 (44.2)	23 (51.1)	0.4
Diabetes, *n* (%)	45 (27.6)	14 (31.1)	0.6
HTN, *n* (%)	82 (50.3)	25 (55.6)	0.5
IHD, *n* (%)	45 (27.6)	14 (31.1)	0.6
Atrial fibrillation, *n* (%)	16 (9.8)	4 (8.9)	0.9
CHF, *n* (%)	14 (8.6)	4 (8.9)	0.9
PVD, *n* (%)	5 (3.1)	1 (2.2)	0.8
CVA, *n* (%)	13 (8.0)	6 (13.3)	0.3
COPD, *n* (%)	21 (12.9)	5 (11.1)	0.7
Cirrhosis, *n* (%)	1 (0.6)	0 (0.0)	0.6
Nephrectomy, *n* (%)	7 (4.3)	2 (4.4)	0.9
BPH, *n* (%)	18 (11)	7 (15.6)	0.4
Smoking, *n* (%)	40 (26.1)	20 (46.5)	0.01
Autoimmune disease, *n* (%)	7 (4.3)	1 (2.2)	0.5
Hydronephrosis history (pre-index), *n* (%)	5 (3.1)	6 (13.3)	0.006
**History of surgery, *n* (%)**			
Major	13 (8)	9 (20)	0.01
Minor	26 (16)	11 (24.4)	0.01

IQR, interquartile range; GU, genitourinary; CKD, chronic kidney disease; eGFR, estimated glomerular filtration rate; AKI, acute kidney injury; EOS, eosinophils; LDH, lactate dehydrogenase; HGB, hemoglobin; WBC, white blood cells; CRP, C-reactive protein; NSAIDS, nonsteroidal anti-inflammatory drug; ACEI, angiotensin-converting enzyme inhibitors; ARB, angiotensin receptor blocker; SGLT2i, sodium-glucose cotransporter-2 inhibitors; PPI, proton pump inhibitors; HTN, hypertension; IHD, ischemic heart disease; CHF, congestive heart disease; PVD, peripheral vessels disease; CVA, cerebrovascular accident; COPD, chronic obstructive pulmonary disease; and BPH, benign prostatic hyperplasia.

**Figure 2:**
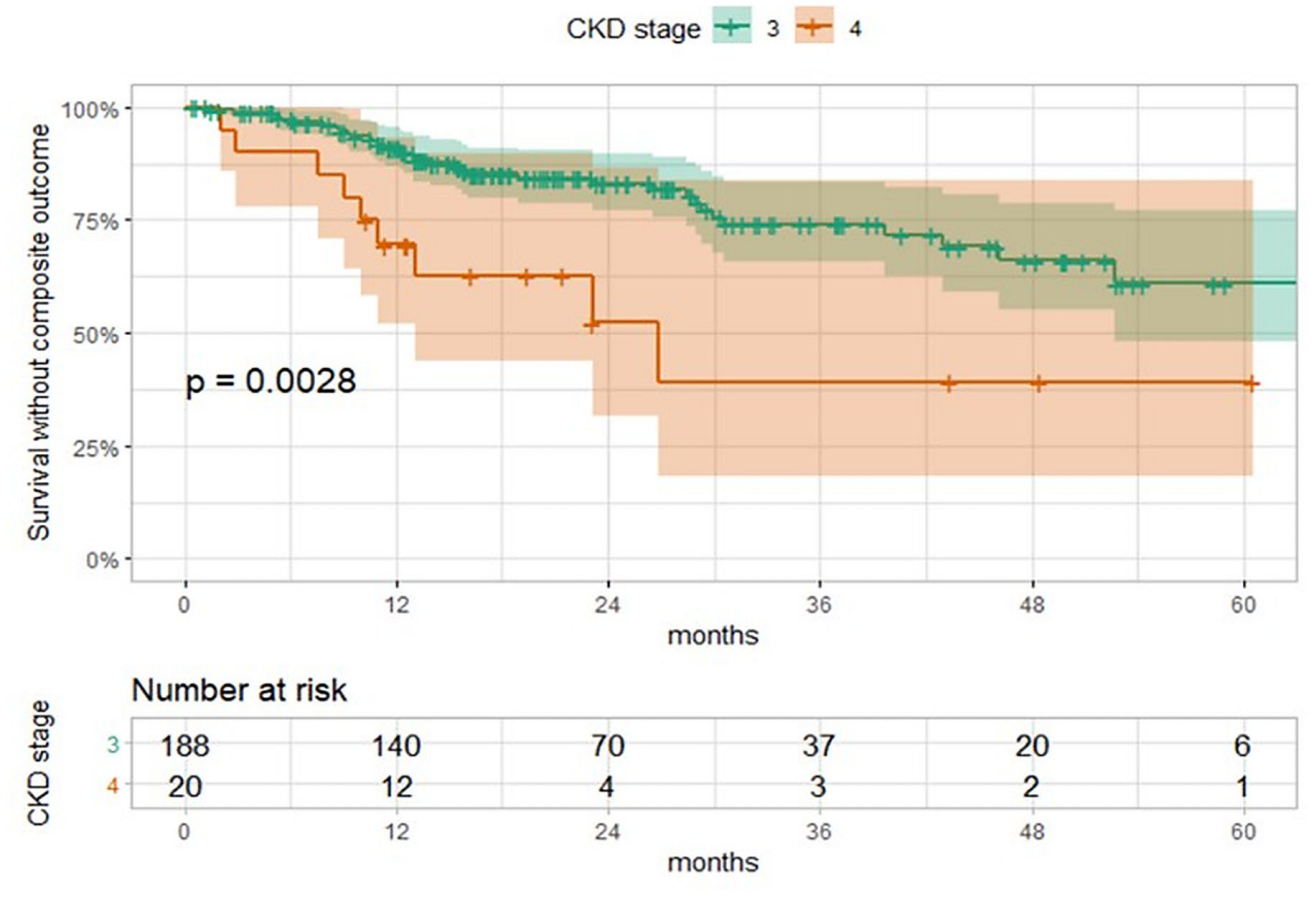
Time to the composite kidney outcome according to CKD stage. Patients with CKD stage 4 reached the composite outcome earlier than those with CKD stage 3 (27 vs. 83 months; *P* = 0.003).

The median time to the composite outcome in the ICI cohort was 83 months. Patients with CKD stage 4 reached the composite outcome earlier than those with CKD stage 3 (27 vs. 83 months; *P* = 0.003) (Fig. 2).

**Figure 3:**
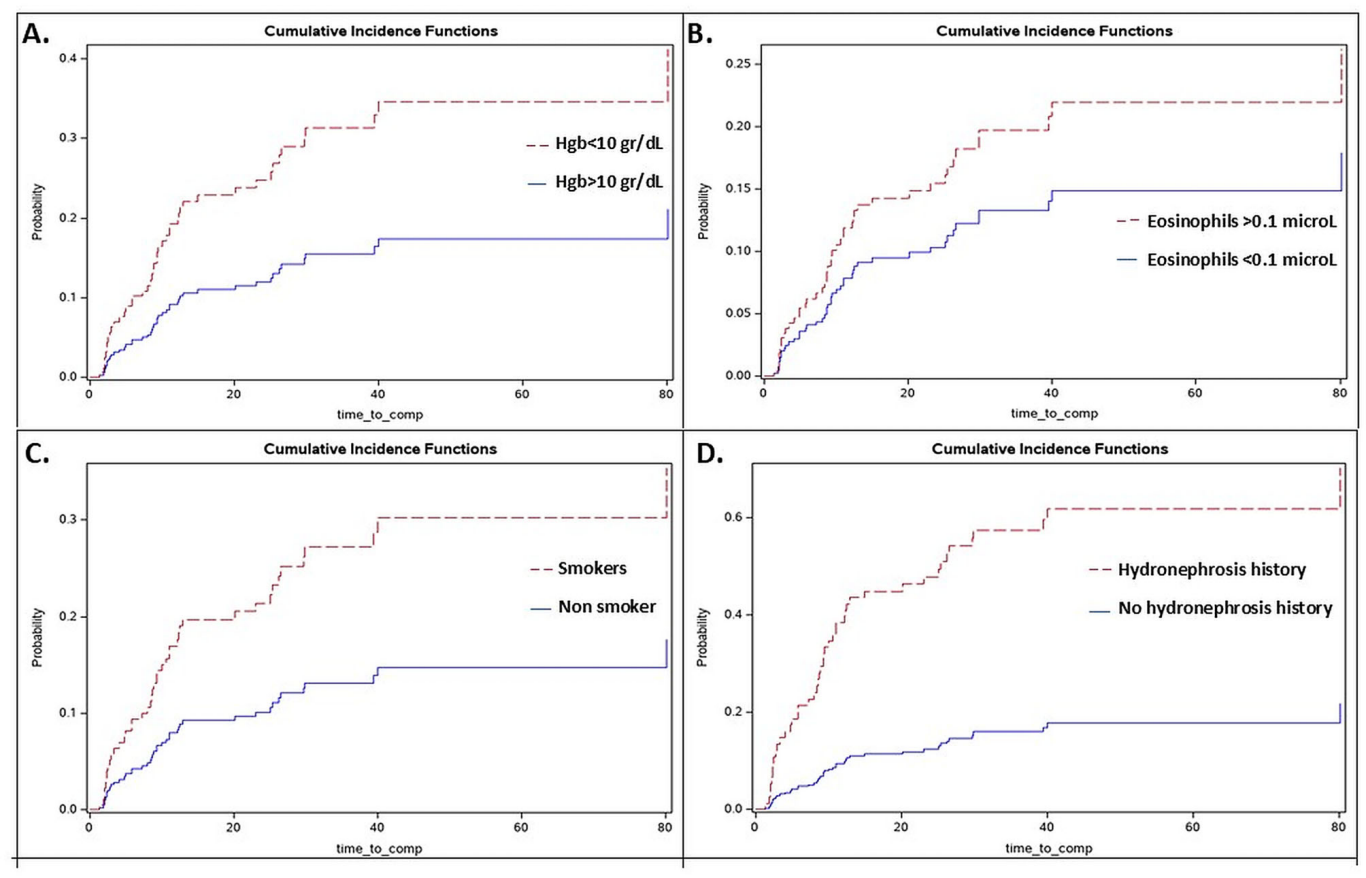
Cumulative incidence of the composite outcome across selected covariates using univariable Fine and Gray models: (a) hemoglobin <10 vs. >10 g/dl; (b) baseline eosinophils >100 vs. <100/µl; (C) smoker vs. non-smoker; and (d) history of hydronephrosis vs. no hydronephrosis.

In the multivariable Fine and Gray model, a history of hydronephrosis was associated with a 5.6-fold higher risk of kidney deterioration. Each 100/µl increase in baseline eosinophil count was associated with a 4.8-fold increased risk, each 1 mg/dl rise in baseline creatinine with an approximately three-fold higher risk, and smoking with about a two-fold higher risk . In contrast, each 1 g/dl increase in baseline hemoglobin was associated with a 12% lower risk. The use of angiotensin-converting enzyme inhibitors (ACE-I) was not associated with a higher risk of the composite outcome (*P* = 0.08) nor with a reduced risk (Table 2). Figure 3 shows the cumulative incidence of the composite outcome across selected covariates, estimated using univariate Fine and Gray models.

**Table 2:** Multivariable Fine and Gray model adjusted for age and sex for the composite outcome, with death treated as a competing risk.

Multivariable Fine and Gray model for the composite outcome		
Baseline variables	HR (95% CI)	*P*
Baseline creatinine (Per 1 mg/dl increase)	3.0 (1.48, 6.08)	0.002
Eosinophils (Per 100/µl increase)	4.86 (2.47, 9.56)	<0.001
Hemoglobin (Per 1 gr/dl increase)	0.88 (0.78, 0.99)	0.04
ACE-i	1.67 (0.94, 2.96)	0.08
Smoking	2.09 (1.19, 3.67)	0.01
Hydronephrosis history	5.65 (2.97, 10.75)	<0.001

ACEi, angiotensin-converting enzyme inhibitors.

**Figure 4:**
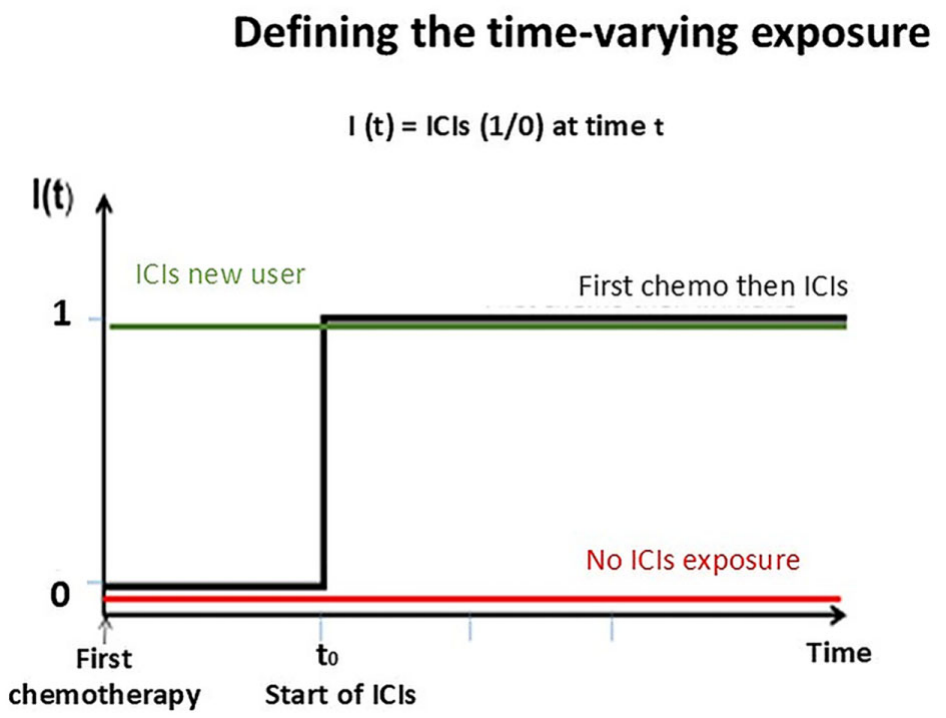
Time-varying exposure definition for ICIs. The red line represents patients who never received ICIs and, therefore, remained unexposed throughout the follow-up period. The black line represents patients who started with chemotherapy alone and later initiated ICIs. The green line represents patients who started ICIs at the first treatment. Overall, 926 patients with an eGFR of 15–60 ml/min/1.73 m² were included in the treatment-comparison analysis: 171 initiated ICIs at the first treatment, 30 switched from chemotherapy to ICIs, and 725 received chemotherapy only. ICIs, immune checkpoint inhibitors; eGFR, estimated glomerular filtration rate.

For the main treatment comparison, 926 patients with a baseline eGFR of 15–60 ml/min/1.73 m² were included: 171 initiated ICIs at the first treatment, 30 started with chemotherapy and later switched to ICIs, and 725 received only chemotherapy (Fig. 4). Seven patients treated with ICIs were excluded because they reached the composite outcome before follow-up began. Median follow-up was 17 months, corresponding to 1815 person-years. ICI exposure was not associated with a higher risk of the composite outcome compared to chemotherapy (HR 0.83, 95% CI 0.58–1.19). Diabetes mellitus and hypertension were similarly distributed between patients who reached the composite kidney outcome and those who did not: diabetes mellitus, 27.6% vs. 31.1% (*p* = 0.60), and hypertension, 50.3% vs. 55.6% (*P* = 0.50). A subgroup analysis of patients with diabetes or hypertension at baseline (528 of the 926 patients included in the main model) yielded similar findings, with an HR of 0.96 (95% CI 0.61–1.52) for immunotherapy vs. chemotherapy.

In the sensitivity analysis, after excluding patients with GU cancers, 710 (77%) patients remained in the model, with 160 events. Results were consistent with the main analysis and indicated that immunotherapy was not statistically significantly different from chemotherapy in risk of the composite kidney outcome (HR = 0.75, 95% CI 0.45–1.25).

To further validate our primary findings and avoid selection bias between treatment groups, we analyzed the propensity score-matched cohort. In this new-user active-comparator analysis, 80 ICI-treated patients were matched 1:1 to 80 chemotherapy-treated controls by age, cancer type, and baseline CKD stage (Table 3). None of the matched chemotherapy controls switched to ICI therapy during follow-up. When comparing these tightly matched cohorts with the marginal Cox model, the results were highly consistent with the primary time-varying analysis. ICI exposure was not associated with an increased risk of the composite kidney outcome compared with chemotherapy (HR 0.81, 95% CI 0.41–1.59).

**Table 3:** Matched groups of new-user active-comparator analysis.

	Group		
Characteristic	80 chemotherapy-new users	80 ICI-new users	*P*
**Age (years), median (IQR)**	74.43 (69.81–78.61)	74.18 (66.55–81.51)	0.9198
**Sex, *N* (%)**			
F	26 (32.5)	22 (27.5)	0.4902
M	54 (67.5)	58 (72.5)	
**Cancer type, *N* (%)**			
Digestive	9 (11.3)	7 (8.8)	0.7855
Genitourinary	5 (6.3)	7 (8.8)	
Lung	39 (48.8)	38 (47.5)	
Skin	20 (25.0)	17 (21.3)	
Other	7 (8.8)	11 (13.8)	
**CKD stage, *N* (%)**			
4	9 (11.3)	6 (7.5)	
3a	53 (66.3)	48 (60.0)	0.3163
3b	18 (22.5)	26 (32.5)	

CKD, chronic kidney disease.

## DISCUSSION

In this real-world group of patients with cancer and baseline eGFR of 15–60 ml/min/1.73 m², 22% of those treated with ICIs reached the composite kidney outcome (new ESKD, initiation of KRT, or a sustained ≥30% decline in eGFR). However, in both the primary time-varying analysis and the new-user active-comparator sensitivity analysis, ICIs were not associated with a higher risk of CKD progression compared with chemotherapy. Importantly, a >30% decline in eGFR indicates a clinically significant high risk of progressing to ESKD. These findings emphasize that a substantial decline in kidney function is common among cancer patients with pre-existing CKD on systemic therapy and should not be viewed as evidence of renal safety of ICIs.

These results suggest that kidney outcomes in this population are mainly influenced by underlying renal vulnerability and individual risk factors rather than by ICI exposure alone. Our findings align with the study by Tiu *et al*. [13], which reported no increased risk of AKI or kidney failure with ICIs in patients with advanced CKD, compared to other anticancer treatments. Our data extend those findings to a wider CKD population with eGFR 15–60 ml/min/1.73 m², focusing on long-term CKD progression rather than only acute renal events. Overall, these results indicate that pre-existing CKD should not automatically exclude patients from receiving ICIs.

The 22% event rate observed in the ICI-only group underscores the high baseline renal risk in this population. Chute *et al*. reported a lower rate of long-term kidney outcomes in a broader group treated with ICIs [23], which likely reflects differences in initial kidney function and case mix. Therefore, our findings should not be interpreted as evidence that ICIs have no effect on the kidneys; rather, they indicate that the risk of renal decline remains significant in patients with CKD undergoing systemic anticancer therapy, whether it involves ICIs or chemotherapy.

We identified hydronephrosis, smoking, higher eosinophil counts, higher baseline creatinine, and lower hemoglobin as factors associated with CKD progression. These are biologically plausible markers of structural kidney disease, systemic illness, and reduced renal reserve [16, 17, 24, 25]. Importantly, our sensitivity analysis excluding patients with genitourinary cancers confirmed that, although structural obstruction (hydronephrosis) significantly accelerates renal decline, the overall risk of CKD progression during systemic therapy persists even in the absence of primary genitourinary malignancies. The connection with eosinophilia is especially noteworthy because eosinophils have been associated with drug-induced interstitial nephritis and immune-related toxicity in ICI-treated patients [12, 26–30]. Although biopsy data were not systematically available, eosinophilia may help identify a subgroup in which immune-mediated injury contributes to renal decline; it might also indicate baseline systemic inflammation or concurrent drug exposure, including proton pump inhibitors [26–30].

AKI was more common among patients who experienced the composite outcome, although the association did not reach statistical significance. The numerical difference suggests that AKI may still be clinically relevant, but our study might have been underpowered to detect an independent effect, given baseline CKD severity and competing risks.

Standard nephroprotective treatments, such as ACE inhibitors and ARBs, were used by a substantial portion of our cohort, and baseline use of these medications did not differ significantly between patients who did and did not reach the composite kidney outcome. Their use was not associated with a lower risk of the composite kidney outcome. Users of these drugs likely had a higher baseline burden of cardiovascular and metabolic comorbidities. This finding should not be interpreted as evidence against cardio-renal protective therapy when it is otherwise indicated. Instead, it underscores the importance of close monitoring in complex oncology patients with CKD. Furthermore, baseline use of SGLT2 inhibitors was notably low (5.8%) in this cohort, likely reflecting the historical study period and the gradual adoption of these agents into routine CKD care, particularly in complex oncology populations. Because only a small number of patients were receiving SGLT2 inhibitors, we were unable to conduct a meaningful subgroup analysis. The role of SGLT2 inhibitors in mitigating long-term decline in kidney function in this oncological population remains a critical area for future prospective research. .

From a clinical standpoint, these results support the use of ICIs in patients with eGFR 15–60 ml/min/1.73 m² when clinically indicated. The main emphasis should be on baseline risk stratification, surveillance of kidney function, and management of modifiable factors such as smoking and anemia, rather than avoidance of ICIs solely because of CKD.

### Strengths and limitations

This study has several strengths, including detailed clinical and laboratory data, a focus on a high-risk CKD population that is often underrepresented in trials, and the use of both time-varying exposure analyses and a new-user active comparator design to minimize treatment allocation and immortal-time biases. A Cox model with a time-varying exposure was used as the primary analytical method to compare immunotherapy and chemotherapy, allowing patients to contribute person-time to each treatment based on their actual treatment sequence. This approach is especially relevant to oncology practice, where treatment lines often change over time, and it helps reduce immortal-time bias by accurately attributing events to the exposure present at the time they occur. Because time-varying models can still be vulnerable to time-dependent confounding, we also performed a new-user active comparator analysis, limited to patients starting either immunotherapy or chemotherapy without prior exposure to the other, with propensity-score matching on key baseline characteristics (age, cancer type, CKD stage, and baseline eGFR). Although matching was successful only for a subset of ICIs users, the hazard ratio from this analysis closely matched that of the primary model, providing reassuring evidence that the absence of an excess ICIs-associated risk is consistent.

Several limitations deserve consideration. First, the retrospective single-center design leaves room for residual confounding despite multivariable adjustment and propensity-score matching. Second, the number of patients with CKD stage 4 was limited, and the findings cannot be generalized to patients with eGFR <15 ml/min/1.73 m². Third, the requirement for at least 1 year of survival may have selected for patients with longer survival and more opportunities for outcome assessment. Fourth, reflecting real-world clinical practice over the 10-year study period, antitumor regimens and dosages were not static. Patients likely underwent dose reductions, treatment delays, discontinuation, or therapy switching based on tolerability, disease course, and evolving oncological standards. Although our retrospective dataset allowed classification of treatment exposure at the regimen level (ICI and/or chemotherapy), it did not consistently capture granular longitudinal details, such as cumulative dose intensity or cycle-by-cycle dose modifications. Therefore, these time-varying treatment changes could not be formally modeled, which represents a limitation of the study. However, unlike classic cumulative-dose nephrotoxicity, ICI-associated kidney adverse events are typically immune-mediated and may occur after variable periods of exposure, with large cohorts reporting a median onset of ∼14–16 weeks after ICI initiation [12, 26].

A key limitation of our retrospective study is the absence of detailed baseline data on proteinuria, CKD etiology, AKI causes and recovery, and kidney biopsy results, which restricts mechanistic understanding. Proteinuria is a strong, independent risk factor for CKD progression, and many antineoplastic drugs may cause or worsen it. Because we lacked baseline and follow-up proteinuria data, we could not adjust for this important confounder.

Furthermore, our dataset did not systematically capture the primary causes of baseline CKD, such as diabetic kidney disease, hypertensive nephrosclerosis, or glomerulonephritis, each of which may be associated with distinct patterns of kidney disease progression. However, none of the patients in our cohort had nephrotic syndrome or documented systemic autoimmune disease with kidney involvement at baseline. The overall clinical profile suggests that CKD was likely related, at least in part, to chronic comorbidities, particularly diabetes mellitus and hypertension. Importantly, these comorbidities were similarly distributed between patients who did and did not reach the composite kidney outcome. The subgroup analysis of patients with diabetes or hypertension at baseline (81% of patients included in the main model) shows an HR of 0.96 (95% CI, 0.61–1.52) for immunotherapy vs. chemotherapy. This suggests that an imbalance in these common CKD-related conditions is unlikely to explain the observed results.

Nevertheless, the absence of detailed data on specific underlying kidney diseases, including primary glomerular disorders, limits mechanistic interpretation and may have introduced residual confounding. This limitation should be considered in the context of broader CKD biology, as progressive kidney disease from different etiologies often converges on shared pathways of tubulointerstitial fibrosis, glomerulosclerosis, tubular atrophy, chronic inflammation, and nephron loss. Therefore, susceptibility to subsequent kidney insults may be influenced not only by the original renal diagnosis but also by baseline renal reserve, fibrosis burden, and comorbidity profile. In addition, the lack of detailed information on AKI causes, recovery patterns, and kidney biopsy findings limited our ability to distinguish true immune-mediated renal injury from other nephrotoxic or hemodynamic causes.

## CONCLUSIONS

Among patients with cancer and baseline eGFR of 15–60 ml/min/1.73 m², ICIs were associated with substantial renal event rates but did not increase the risk of CKD progression compared to chemotherapy. Kidney outcomes appear to be mainly influenced by baseline kidney dysfunction and related clinical risk factors, highlighting the importance of risk assessment, monitoring, and multidisciplinary management during systemic anticancer therapy.

## Data Availability

The data underlying this article are available from the corresponding author on reasonable request and subject to approval by the institutional ethics committee.
